# The Impact of Active Learning Pedagogies on the Self-Efficacy of Neurodiverse Students in Higher Education

**DOI:** 10.1007/s40670-026-02845-x

**Published:** 2026-07-31

**Authors:** Laura Collopy

**Affiliations:** Faculty of Medicine, Imperial College London, National Heart and Lung Institute, Guy Scadding Building, Cale Street, London, SW3 6PT UK

**Keywords:** Neurodiversity, Self-efficacy, Active learning, Inclusive pedagogy, Higher education, STEM

## Abstract

Self-efficacy - the belief in one’s ability to complete a task effectively - is a key determinant of academic success and graduate outcomes in higher education. While active learning enhances engagement and self-efficacy in neurotypical students, little is known about how neurodiverse students experience its social and sensory demands. Grounded in Bandura’s social cognitive theory of self-efficacy, this pilot study explored how active learning impacts the self-efficacy of neurodiverse students in the BSc Medical Biosciences and MSc Genomic Medicine programmes at Imperial College London. An explanatory sequential mixed methods design was employed: a self-efficacy questionnaire provided quantitative breadth, with findings informing subsequent semi-structured interviews that explored the conditions under which self-efficacy was built or undermined. Three themes emerged: connection, control, and balance, corresponding to Bandura’s sources of self-efficacy (vicarious learning and social persuasion, mastery experience, and physiological and emotional states respectively). Participants valued peer and instructor connection and thrived when adequately prepared and in control of their learning. However, self-efficacy was fragile and dynamic, vulnerable to sensory overload and negative feedback. The findings demonstrate that active learning can support neurodiverse students’ self-efficacy, but only when environments are designed to promote psychological safety, predictability, and autonomy. Practical recommendations for neuroinclusive active learning design are offered.

**Supplementary Information:**

The online version contains supplementary material available at https://doi.org/10.1007/s40670-026-02845-x.

## Introduction

Self-efficacy is an individual’s belief in their ability to achieve goals [1]. There is a wealth of evidence showing that it has profound implications for student persistence, motivation, and achievement in higher education [2, 3]. Bandura (1997) identifies four primary sources of self-efficacy: mastery experiences, vicarious learning through social modelling, social persuasion, and physiological or emotional states [4]. Students who experience mastery, achieving success through effort, develop resilience and confidence. This makes self-efficacy a critical factor in academic success and subsequent employment outcomes, as employers seek graduates who are ‘*not only smart and able to learn quickly*,* but also are adaptable*,* responsible*,* and able to work with others’* [5, p. 154].

Neurodivergent students, defined as those whose cognitive profiles diverge from neurotypical norms, often experience lower self-efficacy in educational settings [6]. Neurodiversity encompasses autism, attention deficit and hyperactivity disorder (ADHD), dyslexia, dyspraxia, dyscalculia, and other autism spectrum disorders [7]. Students with autism, ADHD and dyslexia exhibit lower self-efficacy that neurotypical peers and are less engaged in learning [6, 8, 9]. Research has identified numerous challenges encountered by neurodivergent students in higher education settings, including struggles with social and emotional adjustment, and diminished academic performance [10], along with elevated stress and anxiety levels, reduced confidence in their academic abilities, heightened feelings of inadequacy and uncertainty, and more severe tendencies toward self-criticism when compared to their neurotypical peers [11]. Further, neurodiverse students generally report a lack of support and judgemental attitudes from lecturers, when they disclose their learning differences, which contributes to lower self-efficacy [12, 13]. For neurodivergent students, self-efficacy is often shaped by prior educational experiences, social interactions, and perceptions of competence [14, 15]. Those who have faced stigmatisation or misunderstanding in previous settings may enter higher education with diminished confidence in their abilities. In turn, neurodivergent graduates are significantly less likely to find meaningful employment, despite their potential for success [15]. The Association of Graduate Careers Advisory Services (2022) reports that, among disabled graduates in the United Kingdom, autistic graduates have the lowest rate of full-time employment (57.5%), with only around one third employed in roles commensurate with their level of qualification [16].

Active learning pedagogies have demonstrated significant benefits for self-efficacy, engagement, and higher-order thinking [17–19]. Active learning is an interactive pedagogy whereby instructors lead students in self-construction of knowledge [20], requiring students to prepare individually then engage collaboratively to apply their knowledge in solving complex problems [21]. Approaches such as team-based learning (TBL) promote independence and teamwork, creating environments where mastery experiences and social modelling - two key sources of self-efficacy - can flourish. Nevertheless, active learning presumes a level of social confidence and verbal fluency that may not align with the skills of all learners. There is limited research into the impact of active learning on the self-efficacy of neurodiverse students. Berkson et al. (2020) report significant challenges for neurodiverse students during team-based learning, including extreme anxiety that often leads to failure to attend lessons [22]. Wright (2024) reports on emotional distress experienced by neurodiverse students during active learning. However, neither study specifically explores self-efficacy of neurodivergent students, or considers those enrolled in university programmes that utilise active learning for the majority of teaching [23]. This therefore raises a critical question: while active learning increases self-efficacy in neurotypical students, does this benefit extend to neurodivergent learners, or do the social and collaborative demands create barriers that undermine their confidence and engagement?

Between 2020/21 and 2024/25, the number of students in higher education with a known learning difference (e.g. dyslexia, dyspraxia or ADHD) or an autistic spectrum condition increased by 28,470 increasing from 6.7 to 7.9% of the student population [24]. This demands greater understanding of how active learning pedagogies impact their learning experience and self-efficacy.

The four sources of self-efficacy from Bandura’s framework [4] provided a structured lens through which to examine precisely where active learning supports or undermines neurodivergent students’ confidence. They map directly to the conditions that active learning environments create: collaborative problem-solving, peer modelling, instructor feedback, and a socially and sensorially demanding classroom. It is important to acknowledge that self-efficacy is not a stable trait but a dynamic construct, shifting in response to environmental and interpersonal factors. Bandura further extends this to the collective, proposing that groups develop a shared belief in their combined capability (‘collective agency’), which in turn shapes individual confidence [25]. This is particularly pertinent in team-based learning, where stable group membership over time shapes the conditions under which individual self-efficacy is built or eroded.

This pilot study explored the self-efficacy of neurodivergent students through a questionnaire and semi-structured interviews. It was hypothesised that active learning would negatively impact the self-efficacy of neurodivergent students. The theoretical lens described directly informed the design of the self-efficacy questionnaire and the focus of the semi-structured interviews. Results demonstrate that active learning can and does increase self-efficacy in neurodivergent students, but they face unique challenges in the classroom that should be considered by teachers. Given the well-established relationship between self-efficacy and graduate employment outcomes, and the disproportionately low rates of employment among neurodivergent graduates [15, 16], understanding how to cultivate self-efficacy in this growing student population is not merely a pedagogical concern but a social and economic one. Bandura’s framework suggests that small, intentional changes to how active learning is designed and facilitated, ensuring neurodivergent students experience genuine mastery, positive social modelling, and affirming feedback, could have far-reaching consequences for their confidence, resilience, and ultimately their life chances beyond university [4].

## Methods

### Research Design

A pragmatist, mixed methods research design was employed to capture both the breadth and depth of neurodivergent students’ experiences of active learning on the BSc Medical Biosciences and MSc Genomic Medicine courses at Imperial College [26]. These programmes both employ a blended approach to learning. The lessons in both of these programmes are all compulsory and exemplify active learning environments. As such, students are unable to opt out of active learning lessons. An explanatory sequential design was adopted, in which quantitative survey data were collected and analysed first, with findings used to identify themes for subsequent qualitative exploration through semi-structured interviews [26]. This design was chosen because neither method alone could adequately address the research question: the survey enabled systematic measurement of self-efficacy across the cohort, while the interviews provided the interpretive depth needed to understand the conditions under which those beliefs were formed and undermined. A constructivist epistemology informed the qualitative strand, reflecting the view that participants’ accounts of their experiences are contextually situated and individually constructed [27].

### Context and Participants

For this study, neurodivergent students from within the BSc Medical Biosciences (years 1, 2 and 3), or the MSc Genomic Medicine programme were recruited. This is due to the active learning approach adopted across these courses. It is conceivable that, if a student on another course knew about upcoming teamwork and did not wish to take part, they may not attend that class or not take part in a specific exercise. This is not an option for BSc Medical Biosciences, as every class involves group work and most sessions follow, at least to some degree, the traditional TBL format. Similarly, the MSc Genomic Medicine programme makes active group work difficult to avoid. The blended approach requires students to attend a compulsory in-person teaching week per module, where dynamic teaching approaches are employed and students are required to actively engage throughout.

Participants were recruited using criterion sampling, a purposive approach in which individuals are selected on the basis of specific, pre-defined characteristics relevant to the research question [27]. The inclusion criteria were: (1) current enrolment on a programme that uses active learning as its primary pedagogical approach, and (2) self-identification or formal diagnosis of neurodivergence. These criteria ensured that all participants had direct, sustained experience of the phenomenon under investigation [28]. All students in Years 1–3 of the BSc Medical Biosciences course and either full- or part-time students on the MSc Genomic Medicine course at Imperial College London were contacted via email, requesting their participation. The email targeted neurodivergent individuals, diagnosed or self-identified, as there are a number of reasons diagnoses are not sought by individuals, including cultural reasons, with diagnostic criteria also varying in different countries [29]. There is no feasible way to ensure that neurodiversity has been diagnosed using the same parameters in all participants, nor would this be beneficial to the study, so a diagnosis was not required.

### Data Collection

Data were collected in two phases. The first phase was a self-report questionnaire (17 questions, appendix i) measuring self-efficacy on a five-point Likert scale ranging from *strongly disagree (1)* to *strongly agree (5)*. Questions were drawn from Djokic et al. and van Zyl et al. [30, 31]. Both studies originally developed self-efficacy questionnaires for general academic contexts. The surveys were adapted for this study by replacing references to generic academic tasks with references to active learning specifically, for example, substituting ‘*during lectures’* with ‘*during active learning’*. An example item is ‘*I believe that I can successfully participate in discussions during active learning’.* This generated responses from 24 participants (Table 1). Given the small sample size, quantitative analysis was limited to descriptive statistics and is interpreted as exploratory rather than inferential.

**Table 1:** Characteristics of survey respondents (*n* = 24)

Item	Category	Frequency
Gender	Male	6
	Female	17
	Non-binary	1
	Other/prefer not to say	0
Age (years)	18–20	20
	21–25	3
	26–30	1
	31 and older	0
Programme of study	BSc Medical Biosciences Y1	12
	BSc Medical Biosciences Y2	8
	BSc Medical Biosciences Y3	0
	MSc Genomic Medicine FT	4
	MSc Genomic Medicine PT	0
Neurotype	ASD	6
	ADHD	8
	Dyslexia	3
	Dyspraxia	1
	Other	1
	Multiple	2
	Not stated	3

*FT* full-time, *PT* part-time, *ASD* autism spectrum disorder, *ADHD* attention deficit and hyperactivity disorder

The second phase of data collection was the completion of semi-structured interviews with 8 students on a one-to-one basis (Table 2). Students were provided with three vignettes (appendix ii), based on published accounts of neurodiverse student experiences, which were used to structure discussions around their own experiences. Interviews lasted approximately 45-minutes each and were audio-recorded and transcribed verbatim. Due to the limited participant cohort, data is to be considered as a pilot study.

**Table 2:** Questionnaire respondents who volunteered for interview (*n* = 8)

Student ID	Programme of study	Gender	Age (years)	Neurotype
S1	BSc Medical Biosciences Y1	Female	18–20	ASD
S2	BSc Medical Biosciences Y1	Male	18–20	ADHD
S3	BSc Medical Biosciences Y2	Male	18–20	ASD
S4	BSc Medical Biosciences Y1	Male	18–20	ASD & ADHD
S5	BSc Medical Biosciences Y2	Female	18–20	Dyslexia
S6	MSc Genomic Medicine Y1FT	Female	21–25	ASD
S7	MSc Genomic Medicine Y1FT	Male	21–25	ASD
S8	MSc Genomic Medicine Y1FT	Male	21–25	ADHD

*Y* year (of study), *FT* full-time, *ASD* autism spectrum disorder, *ADHD* attention deficit and hyperactivity disorder

Participants were assured explicitly in the recruitment email and participant information sheet that involvement was entirely voluntary, that their responses were anonymous (survey) or confidential (interviews), and that participation would have no bearing on their studies or relationship with the institution. Awareness of the potential for power dynamics to shape responses informed the interview approach, with particular care taken to use open, non-leading questions and to affirm participants’ right to decline to answer or to withdraw.

### Data Analysis

Questionnaire responses were compiled and the mean score and standard deviation calculated for each question. The items were split into 13 positively-worded questions (where a higher score indicated high self-efficacy) and 4 negatively-worded questions (where a higher score indicated low self-efficacy). Cronbach’s alpha was calculated for each group to indicate internal consistency. It was anticipated that the negatively-worded items (Q14–17) might behave differently from the positively-worded items, as reverse-scored items are known to reduce scale coherence in some populations [31]. These items were therefore treated as a separate subscale and interpreted independently rather than as part of a composite score. Emerging themes were then selected for further discussion at interview.

Interview data were analysed using reflexive thematic analysis following the six-phase process described in [28]: familiarisation with the data, generating initial codes, searching for themes, reviewing themes, defining and naming themes, and producing the report. Transcription was followed by open and axial coding, fragmenting data into meaningful phenomena and labelling these to identify patterns across participants. During coding, particular attention was paid to language relating to confidence, self-efficacy, and the emotional and cognitive experience of active learning. Coded extracts were drawn together into candidate themes, which were reviewed iteratively against the full dataset before being refined into the three final themes reported here. The analysis was both theory-informed, guided by Bandura’s self-efficacy framework [4], which shaped initial sensitising codes, and data-driven, allowing emergent insights to extend and nuance theoretical interpretation [28].

### Ethical Considerations

Ethical approval was granted by the Education Ethics Review Process (EERP) at Imperial College London. Participation was voluntary and confidential; no identifiable data were recorded. Interviewees provided written consent and could withdraw at any stage before transcription. Sensitive topics were handled with care, and participants were offered information on university support services if distress arose.

## Results

Results are presented in two phases, consistent with the explanatory sequential design of this study. Survey findings are reported first, providing a quantitative overview of self-efficacy across the cohort. These findings directly informed the thematic focus of the semi-structured interviews, which are reported second. Throughout the interview themes, relevant survey data are referenced to show where quantitative and qualitative findings converge or diverge, and to illustrate what the combined dataset reveals that neither strand could demonstrate alone.

Twenty-four students responded to the anonymous survey (BSc Medical Biosciences (*n* = 20) and MSc Genomic Medicine (*n* = 4)). Eight respondents volunteered for follow-up interviews, as shown in Table 2.

### Survey Findings

#### Active Learning Generally Increases Self-Efficacy in Neurodivergent Students

Questionnaire data indicated that neurodivergent students have a generally positive attitude towards active learning. Questions 1–13 (Table 3, Cronbach α = 0.874) highlighted that students experience higher self-efficacy with regard to working with their peers, versus interacting with teachers and participating in whole-class discussions. For example, the mean Likert score for the question *‘I believe my peers value my contributions during group discussions’* was 3.42, versus a score of 2.63 for the questions ‘*I feel confident in expressing my opinions during whole class discussions’*. One reported at the end of the survey, *‘When I am called on and I have to be reporter I choke on my words because I was most definitely distracted*,* it is hard to stay focused’.* It is also of note that the noise level in the classroom reduced confidence, with a mean score of 2.63.

**Table 3:** Questionnaire scores (from five-point Likert scale) and consistency estimate

	Question	Mean	SD	α
Q1	I believe that I can successfully participate in discussions during active learning.	3.46	0.94	0.874
Q2	I am confident that I can make contributions that are useful to my group during active learning	3.46	0.94	
Q3	I am confident that I can keep up with the pace of group discussions during active learning	3.33	1.15	
Q4	I am confident that I can apply my knowledge to solve problems during active learning, even when I feel under pressure.	3.33	1.12	
Q5	Active learning has INCREASED my confidence.	3.33	1.01	
Q6	I believe that I can successfully take notes during active learning and organise my notes well	3.25	1.03	
Q7	I believe that I can successfully teach other students during active learning lessons.	3.25	0.91	
Q8	I feel confident I can express my opinions during group discussions.	3.21	0.94	
Q9	The motto ‘if other people can, I can too’ applies to me when it comes to active learning lessons.	3.13	0.86	
Q10	I believe that I can ask the teacher to explain a certain part of a lesson which I did not understand again during the lesson.	2.88	0.95	
Q11	I believe that I can successfully answer the teacher’s questions during the lessons	2.88	1.27	
Q12	I feel confident I can express my opinions during whole class discussions.	2.63	1.16	
Q13	I feel confident I can learn with the level of noise in the active learning classroom.	2.63	0.89	
Q14	Active learning causes me anxiety.	2.54	0.98	0.527
Q15	I believe my neurodiversity makes active learning particularly challenging.	4.33	0.73	
Q16	I believe active learning is harder for me than my neurotypical classmates.	4.13	0.76	
Q17	Active learning has DECREASED my confidence.	2.29	1.11	

*SD* standard deviation, α Cronbach’s Alpha

The four ‘negatively worded’ questions (Q14-17, Table 3) notably had a poor Cronbach alpha score (α = 0.527). This is due to the high Likert scores for questions 15 and 16, which indicate that students agree that their neurodiversity does impact their learning and does make active learning more challenging for them than their peers (mean Likert scores of 4.33 and 4.13 respectively, Table 3). Hence, irrespective of the degree of self-efficacy with regard to active learning, neurodivergent students agree that their neurotype plays a significant role in the classroom. It is therefore clear that the way in which these neurodiverse students process their experience of active learning is multifaceted and individualistic. Nonetheless, more participants agreed that active learning had increased their confidence (Q5, Table 3) rather than decreased (Q17, Table 3).

These survey findings generated three areas of focus for the interview phase. The notably lower self-efficacy scores for whole-class discussion and classroom noise (Q12, Q13; mean 2.63) prompted exploration of what conditions undermine confidence. The relatively higher scores for peer interaction (Q1, Q2; mean 3.46) invited deeper examination of how collaboration supports self-efficacy. The high scores for Q15 and Q16, indicating that students strongly believe their neurotype makes active learning more challenging, warranted qualitative exploration of the specific mechanisms through which this occurs across all three themes.

#### Interview Themes

The eight students who provided contact details in their questionnaire responses were contacted for a one-to-one interview (Table 2). Interviews began with discussion of the three vignettes (Appendix ii). Notably, students did not solely relate to one scenario but discussed elements of all three. Following thematic analysis of the interview transcripts, three major themes emerged from the interview data: connection, control, and balance.

#### Connection: Building Self-Efficacy through Collaboration

Contrary to assumptions that teamwork may be distressing for neurodiverse learners, participants emphasised the value of connection during active learning, both with peers and teachers, as key to engagement and self-efficacy. S1 said, ‘*bouncing ideas off your peers can be really helpful. Because if you just like sit down and read any module you might think about it one way*,* but someone else’s way might be different. They can then increase your learning on the topic’.* This discussion of ideas allowed S1 to engage more deeply with the course material, enabling them to feel more confident in their own knowledge and keen to participate in class discussions. This proved to also be the case for others too, with S2 saying, ‘*When I am encouraged to answer on the spot*,* it makes me forced to think*,* and I often realise I know more than I think I do. If I was in the front and the teacher is looking at me*,* it would actually excite me for the teacher to say something and I’d want to respond to that because I feel like we’re thinking the same thing’.* Hence, it is not just the connection with their teammates that students value, but also with their teachers and the wider class. These connections extended beyond academic benefit to emotional reassurance. Students appreciated being known personally by instructors and peers, especially following periods of online learning during the COVID-19 pandemic. S7 affirmed, ‘*Online learning is hard. I like it when someone knows my name. I like it when someone is looking at me to talk’.*

Interviewees emphasised that collaboration with their peers is a key factor in their self-efficacy, across both the BSc and MSc students. S5 reported, ‘*I like that there’s equality within the students and we all have an equal part to play in the group. I feel like we all have an equal responsibility*,* and we should be all be taken seriously’.* Although it is possible that students who did not highly value this connection declined to be interviewed, the importance of connection during active learning was appreciated across all of the participants who were interviewed and is consistent with the survey data, in which items relating to peer interaction produced the highest self-efficacy scores of the positively-worded subscale (Q1 and Q2, mean 3.46, Table 3), suggesting that the value of peer connection is not limited to those who volunteered for interview but is a broader pattern across the cohort. Notably, however, the survey also revealed considerably lower confidence in whole-class discussion (Q12, mean 2.63), indicating that connection is experienced differently depending on the size and formality of the audience, a distinction that the interview data elaborates in detail.

#### Control: Structure as a Foundation for Self-Efficacy

A second theme that arose from the data was that of *control*. Through analysing the data, it became apparent that the efficacy reported by participants fluctuated and was closely linked to control. Their self-efficacy is fragile, and small setbacks can greatly impact them and play on their minds.

Interestingly, several interviewees reported being the loudest and most vocal members during group work at times. However, in all cases, their self-confidence during class relied on how well they had prepared and felt in control of a situation. Their wellbeing in class very much depended on their ability to structure their time beforehand, arrive prepared and essentially know an answer to a question. S6 commented, ‘*I’m really precise with my notes*,* they have to be in a specific way so that I understand them and eModules help me to do it in my own pace and my own time. For me*,* it’s really important to divide tasks in a way that they have*,* like*,* a start an end endpoint. And doing notes in a specific order first helps me to organise and find stuff easier because*,* for example*,* specific parts will have*,* like*,* specific highlighters*,* specific words will have specific underlines. Then if everything is 100% prepared*,* I’m less stressed. I feel more confident and actually more eager to speak out and then probably like be louder’*.

Data revealed that e-learning was key to staying in control, echoing the survey data that highlighted high self-efficacy when it came to taking notes (Q6, Table 3). S4 agreed, ‘*100% e-learning is the best thing because even when I was going to uni and I heard about lectures I thought ‘oh I’m gonna hate it’ because even in during A-levels I never really - I mean I did listen to teacher - but for a lot of the teachers*,* I preferred going from the book instead of from what the teacher was saying because it was really rare that I could fully listen’.*

These data suggest that neurodivergent students like the control that active learning affords them, as they have specific ways they like to work and prepare for class and, if this is achieved, they arrive confident and ready to engage. S1 reported that their ASD caused them to experience communication challenges in social situations, but this did not extend to the classroom where they felt confident in their ability to communicate and interact: ‘*If you met me socially and then academically*,* I would come across as two different people. In an academic group I tend to take the lead most of the time’.*

The preparatory e-learning required on both the BSc and MSc courses in this study, provided autonomy and pacing that supported this need for structure. However, when time constraints prevented preparation, participants reported anxiety and withdrawal from participation. S6 explained, ‘*Last term there were a few weeks where it was really overwhelming with the workload and I wasn’t able to finish my notes in the specific wording and way before the team-based learning*,* and I felt like there were days when I shut down completely and I don’t know. Even if I knew some things because I read through it*,* for example*,* I would just sit quietly because - I don’t even know why*,* I just did’.*

Additionally, the impact of making mistakes was significant and students reported worrying extensively about how they were perceived after getting something wrong. S8 said, ‘*It makes me feel horrible. In my head*,* everything can happen so fast and there is no sense to what I do. It can make me really*,* really nervous and anxious in that moment because others do not know that I know that this doesn’t make sense. And I don’t really want them to think that I’m stupid*,* but I realised that that’s what they must think’.*

Neurodiverse students typically like predictability and routine [32]. It is therefore logical that they value the way they can control their time and the pace of their work and that, when this goes smoothly, they enjoy active learning and the reap the benefits it can offer. However, even seemingly confident students experience stress and anxiety when this control is lost. When speaking about a busy and stressful time of year, S2 said, ‘*There is too much of everything going on*,* so it’s extremely hard to focus. Someone can be talking in the different part of the room and then when 10 people are talking at the same time and you have all these thoughts in your head it’s just like a broken TV. It’s extremely hard to do actually focus on what you’re doing and not lose the attention.*’.

Hence, despite enjoying active learning overall, when workload is heavier and harder to manage, the classroom becomes an intimidating place and the ability to engage can be lost. This additionally reflects the negative impact that classroom noise has on self-efficacy, as reported in the questionnaire (Q13, Table 3).

#### Balance: Emotional Regulation and Cognitive Overload

In the first two themes discussed, neurodivergent students greatly valued *connecting* with staff and peers during active learning, and enjoyed the level of *control* they had learning in this way. However, it was also apparent that much mental energy was required to nurture these connections and also to maintain the level of control they preferred. In trying to balance their efforts, they had good days and bad days. Many references were made to mood and how they felt on a specific day impacted their learning and their ability to work in a team. As discussed above, it was clear from all students that they value connecting with others in class, yet this did not prevent their neurodiversity from impacting the way they connect with others and the stress that this can involve. S7 lamented, *‘I feel like it might be that I’m trying to create like some sort of equilibrium. That really bothers me in a way that*,* not only is it hard for me to work with other people but it is hard for people to work with me*,* which is completely valid and understandable because of how inconsistent I am with the way I work and the way I act. And then you would end up feeling bad. Like ‘oh*,* I’ve been too quiet today. I haven’t really contributed to the work’*,* or ‘Oh no*,* I was too loud and overpowered the conversation’ and it’s just constantly on your mind in this way’*,

S2 self-reports as an extroverted and sociable person and spoke enthusiastically about working with peers. However, they too experienced strong anxiety at times that played on their mind and impacted their relationship with their teammates and their self-efficacy: ‘*Someone in a very abrupt manner said ‘you take a lot of valuable time away from the group and your questions are relevant to the pre reading material*,* so I’d suggest you come better prepared for all of us.’ Which I took offense to because I do try my best in every session’.*

Participants oscillated between confidence and self-doubt, worrying about over- or under-participating. This led to tactics to cope with stress, such as masking or shutting down, with S1 reporting, ‘*If it gets to a point when you’re like experiencing sensory overload when the most little thing just makes your mind go ‘no*,* I can’t do this’*. Such cycles of overthinking, masking, and emotional fatigue reflect the fragility of self-efficacy among neurodiverse students in high-interaction settings.

This fragility is also visible in the survey data. Although the overall Cronbach alpha for the positively-worded subscale was strong (α = 0.874), the wide standard deviations across several items, particularly Q3 (*group discussions move at a manageable pace*, SD = 1.15) and Q11 (*answering teachers’ questions*, SD = 1.27), indicate considerable variability across respondents. This variability is consistent with the interview accounts of oscillating confidence: the same active learning environment that one student experiences as supportive may be experienced as overwhelming by another, or by the same student on a different day. Together, the two datasets suggest that self-efficacy in this population is not only lower on average in certain contexts, but is also substantially less stable than aggregate means alone convey.

Taken together, the survey and interview data offer a more complete account of neurodivergent students’ self-efficacy in active learning than either strand could provide independently. The survey established that self-efficacy is generally positive but uneven, with peer interaction as a consistent strength and whole-class participation and classroom noise as consistent vulnerabilities. The interviews revealed the mechanisms underlying these patterns; the role of preparation and control in enabling mastery, the importance of relational trust in activating vicarious learning and social persuasion, and the emotional cost of managing sensory and social demands that can rapidly erode efficacy beliefs. The integration of both strands confirms that supporting neurodiverse students in active learning requires attention not only to what they can do, but to the conditions under which their belief in that capacity is sustained or lost.

## Discussion

This pilot study sought to understand how active learning impacts the self-efficacy of neurodiverse students within STEM higher education settings. Findings highlight the nuanced experiences of neurodivergent students in active learning environments. Although active learning can promote engagement, collaboration, and deeper learning, its social and sensory demands create both opportunities and vulnerabilities for students with neurodivergent cognitive profiles. Three key, interrelated themes arose: connection, control, and balance. Read through the lens of Bandura’s self-efficacy framework [4], these themes map directly onto his four sources of efficacy belief: connection reflects the operation of vicarious learning and social persuasion within the team environment; control reflects students’ pursuit and preservation of mastery experiences; and balance captures the central, and often precarious, role of physiological and emotional states in mediating self-efficacy under the demands of active learning. Together, the themes illuminate not only whether self-efficacy is promoted or undermined, but through which psychological mechanisms this occurs.

Cognitive Load Theory (CLT) provides a useful context for examining the data in this study. CLT posits that working memory has limited capacity and must balance intrinsic, extraneous, and germane loads to facilitate schema formation [33]. For neurodivergent learners, sensory sensitivity, anxiety, or social processing challenges can contribute to heightened extraneous load, reducing cognitive resources available for learning [32]. In active learning contexts, managing noise, social cues, and time pressures may exacerbate cognitive overload, potentially impacting both engagement and self-efficacy. Indeed, cognitive overload was described by participants in this study as impacting their self-efficacy, leading to feelings of ‘*I can’t do this’.*

Bandura argues that self-efficacy develops partly through *vicarious experience* and *social persuasion*, that is, observing peers succeed and receiving encouragement from others [4]. In this study, participants’ confidence grew through the sense of connection fostered by group activities in an active learning setting. Neurodiverse students valued the opportunity to share ideas, ask questions, and learn from their peers. This reflects collective agency, a core concept in Bandura’s theory, wherein a group’s shared belief in its collective capability enhances individual performance [25]. The findings somewhat contrast earlier research suggesting that teamwork provokes anxiety in neurodiverse learners [22]. The difference may lie in the structure and continuity of the BSc Medical Bioscience and MSc Genomic Medicine programmes. The teams remain stable throughout much of the year, allowing trust and familiarity to develop between students. This structure enables students to engage without constantly renegotiating social dynamics, thereby reducing extraneous cognitive load.

Nevertheless, the benefits of connection were not universal. Some students reported anxiety during whole-class discussions, feeling uncomfortable voicing opinions in front of larger audiences. These findings underscore the need for multiple modes of participation (e.g. verbal, written or digital) to accommodate varying comfort levels and communication preferences, an approach consistent with Universal Design for Learning [34].

The second theme, control, revealed that neurodiverse students’ self-efficacy is closely tied to their sense of preparedness and predictability. When able to structure their learning and control their workflow, participants demonstrated strong engagement and even leadership within their teams. Conversely, when routines were disrupted or time was limited, their confidence declined sharply. This aligns with both self-efficacy theory and cognitive load theory. For students like S6, the ability to organise notes and complete e-learning at their own pace represented a mastery experience that reinforced belief in their competence. This is precisely the kind of incremental, self-directed mastery experience that Bandura identifies as the most robust source of self-efficacy development; one where success is attributable to one’s own effort and preparation rather than luck or external scaffolding [4]. However, high workload and limited preparation time increased *extraneous cognitive load* and emotional stress [33, 35]. The subsequent ‘shutdown’ responses described by participants echo the literature on sensory overload in autism [32, 36].

Importantly, this fragility of control highlights the conditional nature of self-efficacy: it is not a stable trait but a dynamic construct that fluctuates in response to environmental and emotional factors [25]. For neurodiverse students, small disruptions in control, such as unclear instructions, excessive noise, or abrupt scheduling changes, can disproportionately affect their participation.

The final theme, balance, weaves together the interplay of connection and control. Participants described the continuous mental effort required to regulate emotions, interpret social cues, and manage sensory input while contributing meaningfully to teamwork. These findings parallel the literature on masking in neurodiversity, where individuals consciously imitate neurotypical behaviour to fit in, often at the expense of emotional energy and mental wellbeing [37]. Bandura identifies physiological and emotional states as the fourth source of self-efficacy: students draw on their internal emotional condition as evidence of their capability, interpreting anxiety and fatigue as signs of inadequacy and calm as a sign of competence [4]. For the participants in this study, this source was acutely activated. The effort of masking, managing sensory input, and monitoring social participation consumed emotional resources that would otherwise support learning, leaving students in a physiological state that actively undermined their efficacy beliefs. Critically, this dynamic was not always visible to instructors or peers. Students appeared engaged or even confident while simultaneously experiencing significant internal distress.

The pattern described, oscillating between confidence and self-doubt, reveals the cognitive costs of maintaining equilibrium in highly interactive and social learning settings. While active learning offers opportunities for mastery and collaboration, it can also exacerbate stress if the environment lacks psychological safety.

The data presented here suggest a more complex relationship between active learning and self-efficacy than is typically reported in the wider literature. Much of the existing evidence treats active learning as a broadly beneficial intervention: a meta-analysis of 225 studies, found that active learning reliably improved examination performance relative to traditional lecturing [38], while Ballen et al. (2017) reported that science self-efficacy increased for all students following a shift to active-learning pedagogy, regardless of demographic group [39]. In these accounts, active learning functions as a largely unidirectional benefit: students engage more, gain confidence, and perform better as a result, with the environment consistently operating as a net-positive source of mastery and social persuasion in Bandura’s terms.

The present findings complicate this narrative. Rather than a straightforward uplift in self-efficacy, participants described a conditional and, at times, oscillating relationship between active learning and their sense of competence. Where the general literature implies that the mechanisms of active learning act additively to build self-efficacy, the neurodivergent students in this study experienced these same mechanisms as double-edged: the very features that fostered vicarious learning and social persuasion could just as easily produce the anxiety and cognitive overload that undermined their physiological and emotional state. This suggests that active learning’s benefits for self-efficacy, so consistently reported in neurotypical or undifferentiated samples, may not be a stable property of the pedagogy itself, but rather contingent on whether the environment is designed to accommodate diverse sensory and social processing profiles.

Bandura (2008) emphasised the role of emotional state in efficacy development: anxiety undermines perceived competence, whereas calm, supportive environments foster confidence [25]. Educators thus have a crucial role in co-regulating classroom climate, modelling inclusive communication, and normalising diverse participation styles.

### Recommendations

This study offers valuable insights into the mechanisms through which active learning environments can both support and undermine the self-efficacy of neurodivergent students and contribute to a growing body of work advocating for inclusive pedagogy in higher education, particularly within STEM disciplines [15, 40]. Based on the experiences of neurodiverse students on active learning programmes, several practical recommendations emerge:

- Instructors should provide clear schedules, consistent team structures, and advance access to learning materials where possible, as predictability enhances perceived control, a key determinant of self-efficacy.
- Students should be offered multiple ways to contribute to class discussions, to reduce pressure on verbal interaction and promote equitable engagement. Instructors should work to establish explicit norms for respectful dialogue and feedback within teams, modelling vulnerability and empathy to mitigate fear of error.
- Integrating sensory-friendly practices (such as brief, quiet break periods) during active sessions would serve to recognise that managing cognitive and sensory load requires recovery time.
- Training educators in neuroinclusive pedagogies and communication can help reduce unintended barriers and promote belonging.

These strategies align with the Universal Design for Learning (UDL) framework, which advocates proactive design to accommodate learner variability rather than reactive accommodation [34].

## Conclusions

This study provides new insights into the lived experiences of neurodiverse students engaging in active learning within the STEM higher education context. It demonstrates that while neurodiverse students can and do thrive in collaborative learning environments, their self-efficacy is highly contingent on the ability to maintain control and emotional balance. Connection with peers and teachers fosters confidence and belonging, but unpredictability, sensory overload, or negative feedback can quickly erode self-belief.

The findings reinforce that neurodiversity should be understood as *difference*,* not deficit*, echoing the ethos that ‘*The world needs different kinds of minds to work together*’ [41]. Active learning, when designed inclusively, offers precisely this opportunity. However, realising its potential requires a deeper understanding of how neurodiverse students navigate the cognitive and emotional dimensions of teamwork.

Future research should extend this work to larger, more diverse cohorts and explore longitudinal impacts of inclusive active learning on academic outcomes, belonging, and post-graduate trajectories. For now, this study underscores that inclusive design is not only about access, but cultivating environments where all learners can exercise agency, build self-efficacy, and flourish.

### Limitations

An important limitation of this study is that participant self-selection introduces the potential for bias. Students who chose to participate in the survey, and, in particular, those who volunteered for interview, may have had stronger views or more reflective experiences of active learning than those who did not participate. It is plausible that individuals who felt more confident articulating their experiences, or who had more positive or more challenging encounters with active learning, were overrepresented. As such, the findings may not fully capture the perspectives of less engaged or more marginalised students, including those who may avoid participation altogether.

The sample size was relatively small (*n* = 24 for the survey; *n* = 8 for interviews), limiting the statistical power of the quantitative findings and the breadth of perspectives captured qualitatively. While the mixed methods design enabled depth and richness of insight, particularly through the interview data, the sample size constrains the extent to which patterns observed can be considered representative of the wider neurodivergent student population. Further, questionnaire respondents were largely female (17 female vs. 6 male and 1 non-binary), whereas 5 of the 8 interviewees were male. The variability observed in the data further suggests that experiences of self-efficacy in active learning are highly individualised, reinforcing the need for caution in generalisation.

The study was conducted within a single institutional context, focused on two programmes at a research-intensive UK university that employ structured, compulsory active learning approaches. These programmes are characterised by stable teams, significant preparatory e-learning, and a strong emphasis on collaborative problem-solving. As such, the findings may not be directly transferable to other higher education contexts where active learning is implemented differently, is optional, or is less consistently embedded. Institutional culture, class size, disciplinary norms, and levels of pedagogical support are all likely to influence how neurodivergent students experience active learning. Additionally, the students that participated in this study had different levels of active learning exposure due to their age and year of study. It is therefore important to note that survey and interview responses will have been influenced by this experience. Whist this does not detract from the data generated regarding their self-efficacy, the different levels of exposure mean that direct comparisons between participants, and generalisation of specific experiences across the cohort, should be made with caution.

Relatedly, the inclusion of both self-identified and formally diagnosed neurodivergent students reflects an inclusive and pragmatic approach, but also introduces heterogeneity in the sample. Differences in diagnostic status, neurotype, and individual support needs were not controlled for and may shape experiences of self-efficacy in distinct ways. While this reflects the real-world diversity of neurodivergent learners, it limits the ability to draw conclusions about specific subgroups.

## Supplementary Information

Below is the link to the electronic supplementary material.

Supplementary Material 1

## Appendix

### Interview Scenarios

Students that volunteered for the interview section of this study were presented with the following vignettes one week before their interview date. At interview, they were asked to describe which excerpt they identify with the most, and this formed the basis of our semi-structured discussion.

### Student A

*I absolutely hate team-based learning. I can see how useful the activity is for other people. I can see how they would gain from it. I can see if I was able to socialise without the overwhelming anxiety it would be useful for me too. Being with a group of people whom I don’t know is absolutely horrible. These people don’t know me*,* they don’t know how I work. You can’t sit there and try and explain to them while we’re trying to do the work as well. I have been expected to answer questions on the spot in front of the whole class before and I hated it.*

### Student B

*I don’t mind working in a team sometimes*,* I just stay quiet most of the time. I don’t like that the classroom gets noisy and busy with lots of people coming and going. It also frustrates me that my teammates can really affect my learning. The group I was put in had somebody that was quite overpowering. It was not just me that picked up on it*,* the whole group did. She completely shot all my confidence. I can take longer to process information and think about things in a different way so when someone is loud and overpowering it means I don’t get the opportunity to express my opinion.*

### Student C

*I find eModules a good way to learn as I like to print them and can study in my own time. I like to have a bit of paper. But in class*,* everything is on the computer. In class I would prefer to sit right at the front or in the middle. I don’t like to sit with people near me. However*,* in groups we are told where to sit or the group decides where they want to go. I can’t always see the screen how I need to and nothing is printed out for us. If you’re not sat in the right place*,* you can’t see the screen properly*,* you can’t hear properly. I am quite often faced away from the teacher and because of my neurodiversity I get distracted easily by movements and noise. When it comes to group discussions*,* I don’t mind contributing in discussions and having my say*,* but it is much harder for me to follow the lesson.*

## Declarations

### Data availability

Data is available upon request to the corresponding author.

## References

[1] Bandura A. Fearful expectations and avoidant actions as coeffects of perceived self-inefficacy. Am Psychol. 1986;41(12):1389–1391. Available from: 10.1037/0003-066X.41.12.1389

[2] Eccles JS, Wigfield A. Motivational beliefs, values, and goals. Ann Rev Psychol. 2002;53(1):109–132. Available from: 10.1146/annurev.psych.53.100901.135153 11752481

[3] Moussa NM. Promoting Academic achievement: the role of self-efficacy in predicting students’ success in the higher education settings. Psychol Sci Educ. 2023;28(2):18–29. Available from: 10.17759/pse.2023280202

[4] Bandura A. Self-efficacy: The exercise of control. New York: W. H. Freeman; 1997.

[5] Carless D. Learning-oriented assessment: Conceptual bases and practical implications. Innovations Educ Teach Int. 2007;44(1):57–66.

[6] Clouder L, Karakus M, Cinotti A, Ferreyra MV, Fierros GA, Rojo R. Neurodiversity in higher education: a narrative synthesis. Higher Educ. 2020;80:757–778. Available from: 10.1007/s10734-020-00513-6

[7] DANDA (Developmental Adult Neuro-Diversity Association). Neurodiversity. 2011. Available from: http://www.danda.org.uk

[8] Hen M, Goroshit M. Academic procrastination, emotional intelligence. Academic self-efficacy, and GPA: a comparison between students with and without learning disabilities. J Learn Disabil. 2014;47(2):116–124. Available from: 10.1177/0022219412439325 22442254

[9] Sarid M, Lipka O. The relationship between academic self-efficacy and class engagement of self-reported LD and ADHD in Israeli undergraduate students during COVID-19. Eur J Psychol Educ. 2024;39:253–274. Available from: 10.1007/s10212-023-00677-6 PMC9902253 40479354

[10] Frazier TW, Youngstrom EA, Glutting JJ, Watkins MW. ADHD and achievement: meta-analysis of the child, adolescent, and adult literatures and a concomitant study with college students. J Learn Disabil. 2007;40(1):49–65. Available from: 10.1177/00222194070400010401 17274547

[11] Sparks RL, Lovett BJ. College students with learning disability diagnoses: Who are they and how do they perform? J Learn Disabil. 2009;42(6):494–510. Available from: 10.1177/0022219409338746 19704046

[12] Evans D, Granson M, Langford D, Hirsch S. Autism spectrum disorder: reconceptualising support for neurodiverse students in higher education. J High Educ Policy Manag 2023;45(2):243–257. Available from: 10.1080/1360080X.2023.2180168

[13] Mahak S, Malone S, Elsherif M et al. Academic anxiety and cognitive reflection in neurodivergence based on evidence from a large international sample. Sci Rep. 2025;15:37522. Available from: 10.1038/s41598-025-21504-6 PMC12559732 41145747

[14] Griffin E, Pollak D. Student experiences of neurodiversity in higher education: Insights from the autism community. Stud High Educ. 2009;34(3):317–31.

[15] Vincent J. Neurodiversity in higher education: Institutional responses and graduate outcomes. Disabil Soc. 2020;35(3):427–50.

[16] Association of Graduate Careers and Advisory Services. What happens next? 2022. Available from: https://www.agcas.org.uk/write/MediaUploads/Resources/Research%20and%20knowledge/WHN_2022.pdf

[17] Michael J. Where’s the evidence that active learning works? Adv Physiol Educ. 2006;30(4):159–67. 10.1152/advan.00053.2006 17108243

[18] Fook CY, Dalim SF, Narasuman S, Sidhu GK, Fong LL, Keang KM. Relationship Between Active Learning and Self Efficacy Among Students in Higher Education. Int Acad Res J. 2015;1(2):139–49.

[19] Hendrickson P. Effect of active learning techniques on student excitement, interest, and self-efficacy. J Political Sci Educ 2021;17(2):311–325. Available from: 10.1080/15512169.2019.1629946

[20] Hartikainen S, Rintala H, Pylväs L, Nokelainen P. The concept of active learning and the measurement of learning outcomes: a review of research in engineering higher education. Educ Sci. 2019;9(4):276. Available from: 10.3390/educsci9040276

[21] Michaelsen LK, Sweet M. The essential elements of team-based learning. New Dir Teach Learn. 2008;128:7–27.

[22] Berkson L, Richter U, Veerabhatla S, Zasiekina L. Experiences of students with communication related disabilities in the TBL classroom. East Eur J Psycholinguist. 2020;7(1):7–27. Available from: 10.29038/eejpl.2020.7.1.ber

[23] Wright T. How neurodivergence impacts STEM undergraduates in an active learning environment: identifying aspects of active learning that alleviate and/or exacerbate emotional distress and neurodivergent symptomology [Doctoral dissertation]. University of Massachusetts Lowell: ProQuest Dissertations & Theses; 2024. 31637744.

[24] Higher Education Statistics Agency. Who’s studying in HE? Personal characteristics. n.d. Available from: https://www.hesa.ac.uk/data-and-analysis/students/whosinhe/characteristics

[25] Bandura A. An agentic perspective on positive psychology. In: Lopez SJ, editor. Positive psychology: Exploring the best in people. Volume 1. Westport, CT: Praeger; 2008. pp. 167–96.

[26] Creswell JW, Plano Clark VL. Designing and Conducting Mixed Methods Research. 3rd ed. Thousand Oaks, CA: SAGE; 2018.

[27] Savin-Baden M, Howell-Major C. Qualitative research: The essential guide to theory and practice. Routledge; 2013.

[28] Braun V, Clarke V. Using thematic analysis in psychology. Qualitative Res Psychol. 2006;3(2):77–101.

[29] Jurecic A, Neurodiversity. College English. 2007;69(5):421–423. Available from: 10.58680/ce20075864

[30] Djokic S, Brkic-Jovanovic N, Marosan Z, Markovic V. Analysis of academic self-efficacy of medical students learning English for specific purposes. Medicinski Pregled. 2021;74(3–4):74–82. Available from: 10.2298/mpns2104074d

[31] van Zyl LE, Klibert J, Shankland R, See-To EWK, Rothmann S. The general academic self-efficacy scale: psychometric properties, longitudinal invariance, and criterion validity. J Psychoeduc Assess. 2022;40(6):777–789. Available from: 10.1177/07342829221097174

[32] Van Hees V, Moyson T, Roeyers H. Higher education experiences of students with autism spectrum disorder. J Autism Dev Disord. 2015;45(6):1673–88. 10.1007/s10803-014-2324-2 25448918

[33] Sweller J. Cognitive load during problem solving: Effects on learning. Cogn Sci. 1988;12(2):257–85.

[34] Hall TE, Meyer A, Rose DH. Universal design for learning in the classroom: Practical applications. Guilford Press; 2012.

[35] Young JQ, Van Merriënboer J, Durning S, Ten Cate O. Cognitive load theory: Implications for medical education. Med Teach. 2014;36(5):371–84. 10.3109/0142159X.2014.889290 24593808

[36] Richards C, Lipsky D. Managing meltdowns: using the S.C.A.R.E.D calming technique with children and adults with autism. J Autism Dev Disord. 2010;40(7):916–17. 10.1007/s10803-009-0852-y

[37] Krzeminska A, Austin R, Bruyère S, Hedley D. The advantages and challenges of neurodiversity employment in organizations. J Manag Organ. 2019;25(4):453–463. Available from: 10.1017/jmo.2019.58

[38] Freeman S, Eddy SL, McDonough M, Smith MK, Okoroafor N, Jordt H, Wenderoth MP. Active learning increases student performance in science, engineering, and mathematics. Proc Natl Acad Sci USA. 2014;111(23):8410–8415. Available from: 10.1073/pnas.1319030111 PMC4060654 24821756

[39] Ballen CJ, Wieman C, Salehi S, Searle JB, Zamudio KR. Enhancing diversity in undergraduate science: self-efficacy drives performance gains with active learning. CBE Life Sci Educ. 2017;16(4):ar56. Available from: 10.1187/cbe.16-12-0344 PMC5749958 29054921

[40] Partington A. Developing inclusive pedagogies in HE through an understanding of the learner-consumer: promiscuity, hybridisation, and innovation. Post-Digital Sci Educ. 2021;3:102–119. Available from: 10.1007/s42438-020-00110-x

[41] Grandin T. The world needs all kinds of minds [Video]. TED; 2010.

