# Supplementary material for: The Impact of Active Learning Pedagogies on the Self-Efficacy of Neurodiverse Students in Higher Education
Source: Med Sci Educ. 2026 Jul 31;36(5):2381–92. doi: 10.1007/s40670-026-02845-x (PMC13723656; doi:10.1007/s40670-026-02845-x)
Supplement: Supplementary file 1 [file 40670_2026_2845_MOESM1_ESM.pdf]

Questionnaire: neurodiversity and active learning

INFORMATION SHEET FOR PARTICIPANTS

The impact of active learning on the self-efficacy of neurodiverse students

We would like to invite you to participate in this research project. You should only participate if you want to; choosing not to take part will not disadvantage you in any way.

Before you decide whether you want to take part, it is important for you to understand why the research is being done and what your participation will involve. Please take time to read the following information carefully and discuss it with others if you wish. Ask us if there is anything that is not clear or if you would like more information.

This research aims to investigate how the active learning elements of the BSc Medical Bioscience (BMB) and MSc Genomic Medicine course impact how neurodiverse students believe in their own abilities. Students currently enrolled on either course are being recruited.

If you agree to take part, you will complete a questionnaire that asks questions on your experience of active learning. This should take no more than 15 minutes.

The questionnaire can be submitted anonymously so it cannot be traced back to you. You will be given the option to include your contact details, if you are happy to be contacted for an interview on this topic. It is your choice whether to include your contact details or not, and the only person that will see your contact details is the principal investigator.

When your questionnaire has been submitted, your contact details will be removed (if included) and stored separately so your questionnaire data remains anonymous. You will not be identifiable to any third party and the data you provide will not be traceable back to you.

Participation in this study may result in beneficial changes to teaching practice on the BMB or Genomic Medicine course. Publication of the project may be sought.

Submission of a completed (or partially completed) questionnaire (by pressing the 'submit' or 'finish' buttons if online questionnaires are used) implies consent to participate, and for all data collected to be used.

As participation is anonymous it will not be possible to withdraw your data once you have returned your questionnaire. It is your choice whether or not to take part. If you decide to take part you are still free to withdraw from the study by not clicking 'submit'.

If you have any questions or require more information about this study, please contact the researcher using the following contact details:

l.collopy@imperial.ac.uk
Thank you for taking the time to read and consider this information

1. Please state which programme are you enrolled, your year of study and whether you are a full or part-time student.

2. What is your age in years?

- ◯ 18-20
- ◯ 21-25
- ◯ 26-30
- ◯ 31 and over

3. How do you identify your neurotype?

4. Which gender do you identify as?

- ◯ Female
- ◯ Male
- ◯ Non-binary
- ◯ Prefer not to say

5. I believe that I can successfully participate in discussions during active learning.

1	2	3	4	5

Strongly disagree — Strongly agree

6. I am confident that I can make contributions that are useful to my group during active learning

1	2	3	4	5

Strongly disagree — Strongly agree

7. I am confident that I can keep up with the pace of group discussions during active learning

1	2	3	4	5

Strongly disagree — Strongly agree

8. I am confident that I can apply my knowledge to solve problems during active learning, even when I feel under pressure.

1	2	3	4	5

Strongly disagree Strongly agree

9. Active learning has INCREASED my confidence.

1	2	3	4	5

Strongly disagree Strongly agree

10. I believe that I can successfully take notes during active learning and organise my notes well

1	2	3	4	5

Strongly disagree Strongly agree

11. I believe that I can successfully teach other students during active learning lessons.

1	2	3	4	5

Strongly disagree Strongly agree

12. I feel confident I can express my opinions during group discussions.

1	2	3	4	5

Strongly disagree Strongly agree

13. The motto 'if other people can, I can too' applies to me when it comes to active learning lessons.

1	2	3	4	5

Strongly disagree Strongly agree

14. I believe that I can ask the teacher to explain a certain part of a lesson which I did not understand again during the lesson.

1	2	3	4	5

Strongly disagree Strongly agree

15. I believe that I can successfully answer the teacher's questions during the lessons

1	2	3	4	5

Strongly disagree Strongly agree

16. I feel confident I can express my opinions during whole class discussions.

1	2	3	4	5

Strongly disagree Strongly agree

17. I feel confident I can learn with the level of noise in the active learning classroom.

1	2	3	4	5

Strongly disagree Strongly agree

18. Active learning causes me anxiety.

1	2	3	4	5

Strongly disagree Strongly agree

19. I believe my neurodiversity makes active learning particularly challenging.

1	2	3	4	5

Strongly disagree Strongly agree

20. I believe active learning is harder for me than my neurotypical classmates.

1	2	3	4	5

Strongly disagree Strongly agree

21. Active learning has DECREASED my confidence.

1	2	3	4	5

Strongly disagree Strongly agree

22. Would you be willing to take part in an interview lasting 30-45 minutes about your experience as a neurodiverse student on an active learning course? If yes, please provide your email address below. This will be seen only by the Principal Investogator. If you change your mind at a later date you can withdraw from the study.

This content is neither created nor endorsed by Microsoft. The data you submit will be sent to the form owner.

Microsoft Forms
